# Metabolomics profiles in acute-on-chronic liver failure: Unveiling pathogenesis and predicting progression

**DOI:** 10.3389/fphar.2022.953297

**Published:** 2022-08-19

**Authors:** Guofeng Liu, Xiaoze Wang, Xiaoli Fan, Xuefeng Luo

**Affiliations:** Department of Gastroenterology and Hepatology, Sichuan University-University of Oxford Huaxi Joint Centre for Gastrointestinal Cancer, West China Hospital, Sichuan University, Chengdu, China

**Keywords:** ACLF (acute-on-chronic liver failure), OxPhos, systemic inflammation, metabolomics, metabolites

## Abstract

Acute-on-chronic liver failure (ACLF) usually develops based on acute decompensation (AD) of cirrhosis and is characterized by intense systemic inflammation, multiple organ failure, and high short-term mortality. Validated biomarkers for the diagnosis and prognosis of ACLF remain to be clarified. Metabolomics is an emerging method used to measure low-molecular-weight metabolites and is currently frequently implemented to understand pathophysiological processes involved in disease progression, as well as to search for new diagnostic or prognostic biomarkers of various disorders. The characterization of metabolites in ACLF has recently been described via metabolomics. The role of metabolites in the pathogenesis of ACLF deserves further investigation and improvement and could be the basis for the development of new diagnostic and therapeutic strategies. In this review, we focused on the contributions of metabolomics on uncovering metabolic profiles in patients with ACLF, the key metabolic pathways that are involved in the progression of ACLF, and the potential metabolite-associated therapeutic targets for ACLF.

## Introduction

Acute-on-chronic liver failure (ACLF) is a distinct disease state in patients with advanced chronic liver disease characterized by liver failure due to an acute hepatic injury in an underlying chronic liver disease with high 28-days mortality (Sarin et al., 2019). ACLF mainly progresses from acute decompensation (AD) of cirrhosis, which refers to the occurrence of ascites, encephalopathy, gastrointestinal hemorrhage, or any combination of these disorders in patients with cirrhosis (Trebicka et al., 2020). Bacterial infections, alcoholism, and chronic viral hepatitis relapse are the most common precipitating factors in half of patients with ACLF; the remaining patients do not have an identifiable trigger (Moreau et al., 2013; Shi et al., 2015). Patients with ACLF present intense systemic inflammation and multiple organ failures (liver, kidney, brain, coagulation, circulation, and respiration), leading to high short-term mortality (Sarin and Choudhury, 2016; Arroyo et al., 2020). Accumulating evidence has shown that systemic inflammation and immune paralysis are considered the main drivers of extensive tissue injury and organ failure in patients with AD who develop ACLF (Clària et al., 2016; Trebicka et al., 2019). Systemic inflammation has been reported to result mainly from the activation of innate immune cells by pathogen-associated molecular patterns (PAMPs) or damage-associated molecular patterns (DAMPs) (Fernández et al., 2018; Aizawa et al., 2020). However, the underlying mechanisms of ACLF at the tissue and cellular levels that lead to the occurrence and maintenance of organ failures remain to be elucidated. It is crucial to uncover the pathogenesis of ACLF to establish reliable biomarkers to determine the progression and/or regression of ACLF in response to a given treatment.

Metabolomics is a potent approach for measuring the products of individual protein expression, based on comprehensive monitoring of metabolites (mass <1,000 Da) in biological systems (Johnson et al., 2016). The changes at the genome or proteome level of the organism reflect the tendency for specific behaviors of biological systems, while the changes in metabolites embody the current condition of the organism (Bujak et al., 2015). Subtle changes at the genome or proteome level can be amplified due to the higher sensitivity of metabolomics. Moreover, the small molecular chemicals measured by metabolomics are downstream from the genome, transcriptome, and proteome, providing a highly integrated profile of biological status relative to other omics (Newgard, 2017). Owing to metabolome consisting of thousands of different chemical classes, metabolomic measurements are more difficult than genome or proteome. Additionally, the identification of metabolites remains a major challenge in metabolomics due to inadequate databases (Djoumbou Feunang et al., 2016; Wishart et al., 2018).

Metabolomics is mainly divided into targeted and non-targeted metabolomics. Targeted metabolomics focus on identifying and quantifying specific compound classes or metabolic pathways. Untargeted metabolomics aims to include all the metabolites (Jacob et al., 2019). Various analytical techniques are applied in both targeted and untargeted metabolomic studies. Liquid chromatography coupled with mass spectrometry (LC-MS) is widely used owing to the extensive availability and continuous advancement of instruments, while nuclear magnetic resonance (NMR) is suitable for detecting the compounds which contain the hydrogen atoms (Jang et al., 2018). By virtue of continuous advancement in sensitive analysis techniques and progressive biostatistics, metabolites in blood, urine or other body fluids can be determined and identified (Johnson et al., 2016). Changes in metabolites may appear earlier than specific symptoms in diseases. Hence, metabolomics is commonly utilized to search for new diagnostic and prognostic disease biomarkers and to gain a deeper understanding of the pathogenesis of various diseases (Gowda et al., 2008; Mamas et al., 2011).

Recently, the concept that the combined effect of cytokines/chemokines and metabolite-derived factors that act on metabotoxins contributes to systemic inflammation and tissue/organ injury of ACLF has been reinforced. It is noteworthy that metabolomics has been used in ACLF research to characterize ACLF metabolic changes and to identify metabolic signatures as prognostic indicators (McPhail et al., 2016; Moreau et al., 2020; Clària et al., 2021). These studies highlight the intrinsic biological activity of metabolites and provide new ideas for the application of metabolomics, as they can be used to identify metabolites that act as drivers or mediators of biological processes, and thus better understand their physiological role in the progression of ACLF.

In this review, we summarize the pivotal metabolic pathways involved in ACLF that have been identified by metabolomics and assess the use of metabolomics to identify biomarkers related to ACLF.

## Key metabolic pathways associated with ACLF

**FIGURE 1:**
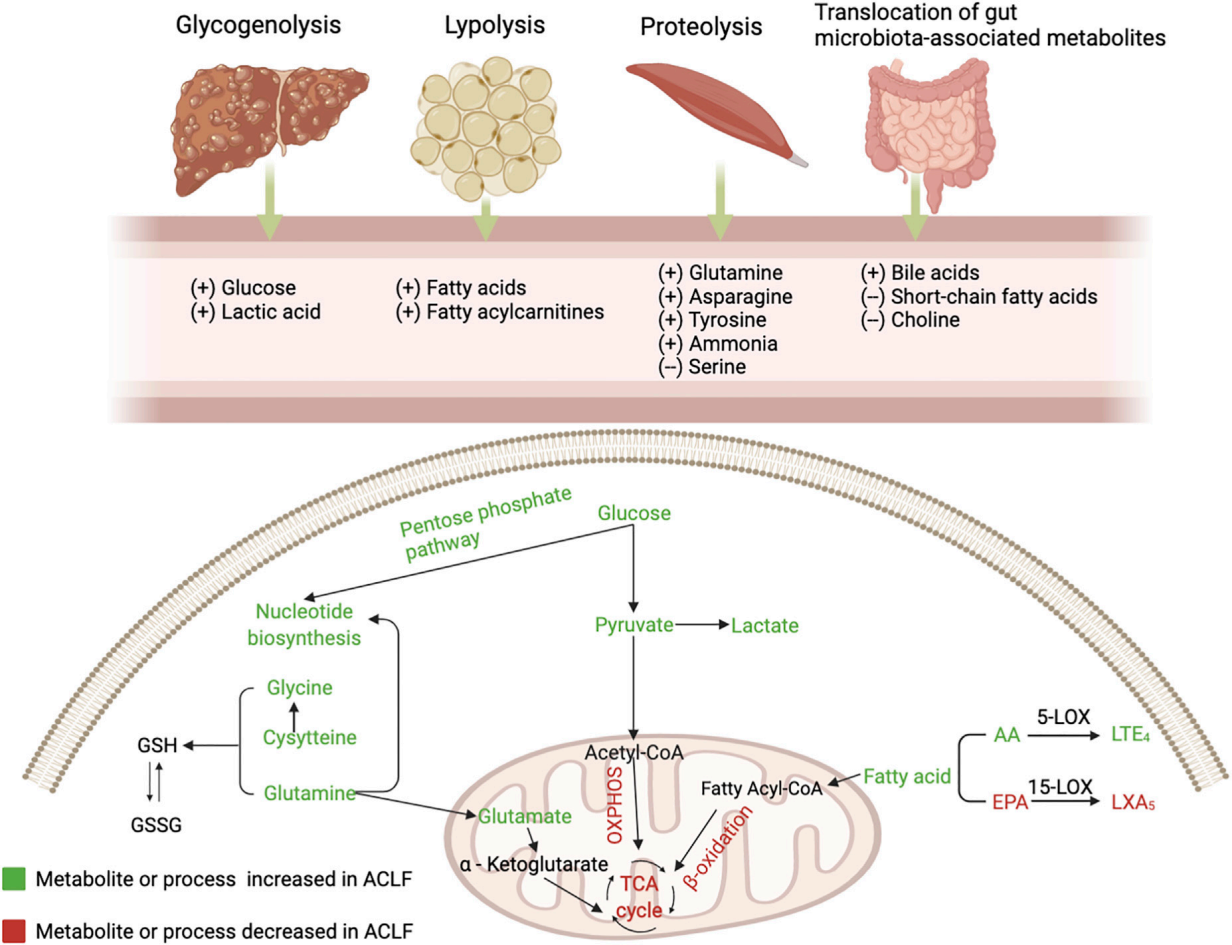
The alterations of serum metabolites and key metabolic pathways in acute-on-chronic liver failure (ACLF). Glycogenolysis (liver), proteolysis (mainly muscles), lipolysis (adipose tissue), and bacterial translocation (gut) are induced in the context of ACLF, resulting in the release of glucose, amino acids, fatty acids and gut microbiota-associated metabolites. During PAMP-induced systemic inflammation, glucose is used for promptly producing ATP through glycolysis and enters the pentose phosphate pathway that is involved in nucleotide synthesis, while mitochondrial oxidative phosphorylation (OXPHOS) is suppressed; Mitochondrial β-oxidation of FAs is inhibited and blood level of fatty acylcarnitines is increased; Increased generation and accumulation of many blood amino acid metabolites are involved in various amino acid metabolism. AA, arachidonic acid; EPA, eicosapentaenoic acid; GSH, reduced glutathione; GSSG, oxidized glutathione; LOX, lipoxygenase; LT, leukotriene; LX, lipoxin; OXPHOS, oxidative phosphorylation; TCA, tricarboxylic acid.

Various metabolic pathways are altered in ACLF due to systemic inflammation, stimulating the hypothalamic-pituitary-adrenal axis and sympathetic nervous system to activate glycogenolysis, proteolysis, and lipolysis. Herein, we review several key metabolic pathways involved in the progression of ACLF (Figure 1).

### Inhibition of oxidative phosphorylation

The liver is the main organ that regulates various pathways controlling glucose metabolism, including glycogenesis, glycogenolysis, glycolysis, and gluconeogenesis (Nordlie et al., 1999). ACLF is characterized by changes in glucose catabolism through specific extramitochondrial pathways (Moreau et al., 2020). The main site of glucose metabolism is transferred to the cytoplasm from the mitochondria in ACLF (Zhang et al., 2021). In the context of systemic inflammation, innate and adaptive immune cells are stimulated by PAMPs or DAMPs. Glucose is distributed to immune cells to maintain energy anabolism and the immune response, which activates intracellular glycolysis pathways by activating key enzymes associated with glycolysis to rapidly generate adenosine triphosphate (ATP) (Van Wyngene et al., 2018; Zhang et al., 2021). Furthermore, mitochondrial OXPHOS is inhibited, partly due to suppression of the electron transport chain by excessive nitric oxide (Van Wyngene et al., 2018; Wang et al., 2019; Moreau et al., 2020). In addition, inhibition of OXPHOS is also associated with increased generation of reactive oxygen species (ROS) (Chan, 2006). The source of mitochondrial OXPHOS is varied, while glycolysis relies on glucose as the single energy source (Mills et al., 2017). Hence, it is less efficient at producing ATP in ACLF.

### Extramitochondrial pathways

Glucose can be involved in the pentose phosphate pathway, contributing to the production of ribose and nicotinamide adenine dinucleotide phosphate (NADPH). Increased ribose promotes nucleotide synthesis, which leads to the production of inflammatory cytokines. NADPH, a hydrogen carrier, is involved in various anabolic reactions and can also produce ROS, further inhibiting mitochondrial OXPHOS (O'Neill and Pearce, 2016).

Zhang et al. (Zhang et al., 2021) indicated that extramitochondrial pathways, including glycolysis, glycogenolysis, and pentose phosphate pathway, were excessively activated in the peripheral blood mononuclear cells (PBMCs) from patients with AD and ACLF. Moreover, the nitrogen and carbon sources were utilized exceedingly in PBMCs from ACLF. Additionally, another metabolic characteristic of PBMCs from AD and ACLF patients is the impaired pyruvate decarboxylation to acetyl-CoA, a process that is intermediated by the pyruvate dehydrogenase complex (PDC) (Zhang et al., 2021). PDC, a multi-enzyme complex located in the mitochondrial matrix, plays a key role in connecting glycolysis with the tricarboxylic acid (TCA) cycle (Reed, 1981). Accordingly, the impaired PDC may partly explain the reduction in OXPHOS in ACLF (Zhang et al., 2021). Generally, disturbance of glycometabolism in leukocytes may further enhance the inflammatory response in patients with ACLF. Indeed, energy production from glycolysis can not be maintained for the long term in advanced chronic liver disease (Nishikawa et al., 2014). Thus, how energy produces remains to be elucidated with the development of ACLF.

### Kynurenine pathway

Pro-inflammatory cytokines, such as interferon (INF)-γ or tumor necrosis factor (TNF)-α, induce tryptophan degradation through the kynurenine pathway, and metabolites of this pathway are involved in the pathogenesis of inflammatory diseases and cancer (Cervenka et al., 2017). It has been confirmed that kynurenine pathway (KP) activation is associated with ACLF development and is also an independent predictor of short-term death in patients with ACLF (Clària et al., 2019). Notably, the lower concentrations of kynurenate and xanthurenate of KP in patients treated with simvastatin and rifaximin indicate an inhibition of this metabolic pathway (Pose et al., 2021).

### Ketone bodies and the ammonia pathway

Amino acids act as nutrients and intracellular signaling molecules, regulating key metabolic pathways that are essential for cellular activity (Baker, 2009). In the context of systemic inflammation, substantial inflammation-linked molecules and acute phase proteins are produced by activated leukocytes and hepatocytes, respectively (Moreau et al., 2013; Clària et al., 2016). The production of these molecules involved in the inflammatory response may be based on the mobilization of a large number of proteinogenic amino acids from skeletal muscle (Zaccherini et al., 2021). Ketone bodies, as fuel for tissues and organs in the body, are produced from fatty acids and ketogenic amino acids (Ganeshan et al., 2019). In ACLF, the production of ketone bodies in the liver relies on the catabolism of ketogenic amino acids due to the inhibition of fatty acid β oxidation (Ganeshan et al., 2019; Moreau et al., 2020). Furthermore, the transsulfuration pathway, a part of the methionine cycle that is involved in one-carbon metabolism, is activated—contributing to the synthesis of glutathione to resist systemic oxidative stress in ACLF (Sanderson et al., 2019).

Circulating ammonia, mainly derived from amino acids through phosphate-activated glutaminase, is increased in decompensation and ACLF (Sawhney et al., 2016). Portosystemic shunts and reduced activity of the urea cycle weaken ammonia removal capacities, leading to hyperammonaemia (Wright et al., 2011). Elevated ammonia not only causes hepatic encephalopathy but is also associated with death and organ failures (Poh and Chang, 2012; Shalimar et al., 2019). Ammonia can serve as a cytotoxic product by changing the cell membrane potential and Pondus Hydrogenii (PH) and producing several free radicals, which leads to mitochondrial dysfunction and aggravates organ failure (Rose et al., 2005; Dasarathy et al., 2017). Furthermore, hyperammonemia can aggravate portal pressure by stellate cell contraction and can directly impair neutrophil phagocytosis, contributing to systemic inflammation (Shawcross et al., 2008; Jalan et al., 2016).

### Fatty acylcarnitines and the sphingolipids pathway

Immune-metabolic dysregulation and inflammation can be mediated by changes in lipid composition (Ertunc and Hotamisligil, 2016). Levels of a large series of fatty acids are increased in plasma due to lipolysis in adipose tissue due to systemic inflammation (Van Wyngene et al., 2018). The liver plays a key role in the synthesis of endogenous lipids (Eisenberg and Levy, 1975). Accordingly, suppression of serum lipid levels is largely attributed to the disturbance of liver function.

There is a reduction in fatty acids (FAs) catabolism through mitochondrial β-oxidation in ACLF(25). Increased levels of fatty acylcarnitines in plasma indicate that mitochondrial β-oxidation is inhibited in peripheral organs in ACLF, partly resulting from the suppression of translocation of fatty acylcarnitines into the mitochondrial matrix mediated by the peroxisome proliferator-activated receptor (PPAR)-α (Ganeshan et al., 2019; Moreau et al., 2020). Ultimately, a large amount of FAs, which cannot be dissimilated via β-oxidation, together with ROS produced by systemic inflammation, causes mitochondrial damage, which contributes to the progression of extrahepatic organ failure (Moreau et al., 2020). Sphingolipids are basic components of cytomembranes and are involved in immunomodulation and maintenance of cell survival (Hannun and Obeid, 2018). Reduced intermediates involved in sphingolipid synthesis and downregulated genes coding for enzymes involved in sphingolipid biosynthesis partly explain the inhibition of the sphingolipid pathway in ACLF (Clària et al., 2021). Paradoxically, a multicenter study from the Chinese Group for the Study of Severe Hepatitis B (COSSH) demonstrated that sphingolipid metabolic pathways were upregulated in PBMCs from acute hepatitis B virus-related acute-on-chronic liver failure (HBV-ACLF), which could be partly explained by different etiologies (Li et al., 2021).

### Bile acids pathway

Increased serum bile acid levels indicate deterioration of liver clearance and the formation of portosystemic shunting (Krähenbühl and Reichen, 1988). Bile acids can exert vasoactive effects and induce splanchnic vasodilation through stimulation of the G protein-coupled bile acid receptor one and can contribute to the development of portal hypertension, which further aggravates the translocation of components of the intestinal flora components translocation (Thomas et al., 1991; Fryer et al., 2014). In addition, bile acids directly alter the gut barrier function by downregulating the nuclear receptor farnesoid-X-receptor (FXR) (Sorribas et al., 2019). Furthermore, bile acids can stimulate the generation of inflammatory cytokines directly in hepatocytes during cholestasis by activating inflammatory pathways, involved in the progress of ACLF (Allen et al., 2011; Horvatits et al., 2017).

### Short-chain fatty acids pathway

Short-chain fatty acids (SCFAs), gut microbiota-derived metabolites that participate in maintaining the integrity of intestinal mucosal barrier and host’s immune response, are markedly reduced in advanced stages of liver disease, while decreased SCFAs are inversely correlated with the severity of portal hypertension, systemic inflammation, and the prevalence of decompensating events (Juanola et al., 2019). Furthermore, reduced SCFAs can affect energy metabolism in decompensated cirrhosis because fewer SCFAs are transformed into acetyl coenzyme A to produce ATP (Crawford et al., 2009). The microbial metabolites of phenylalanine and tyrosine, which engage the aryl hydrocarbon receptor (AhR) and promote interleukin (IL)-22 secretion, and contribute to local immunization, were reported to be reduced in patients who developed ACLF, which manifested a relative immunodeficiency (Gao et al., 2018; Bajaj et al., 2020).

## Biomarkers associated with the development of ACLF based on metabonomics

**FIGURE 2:**
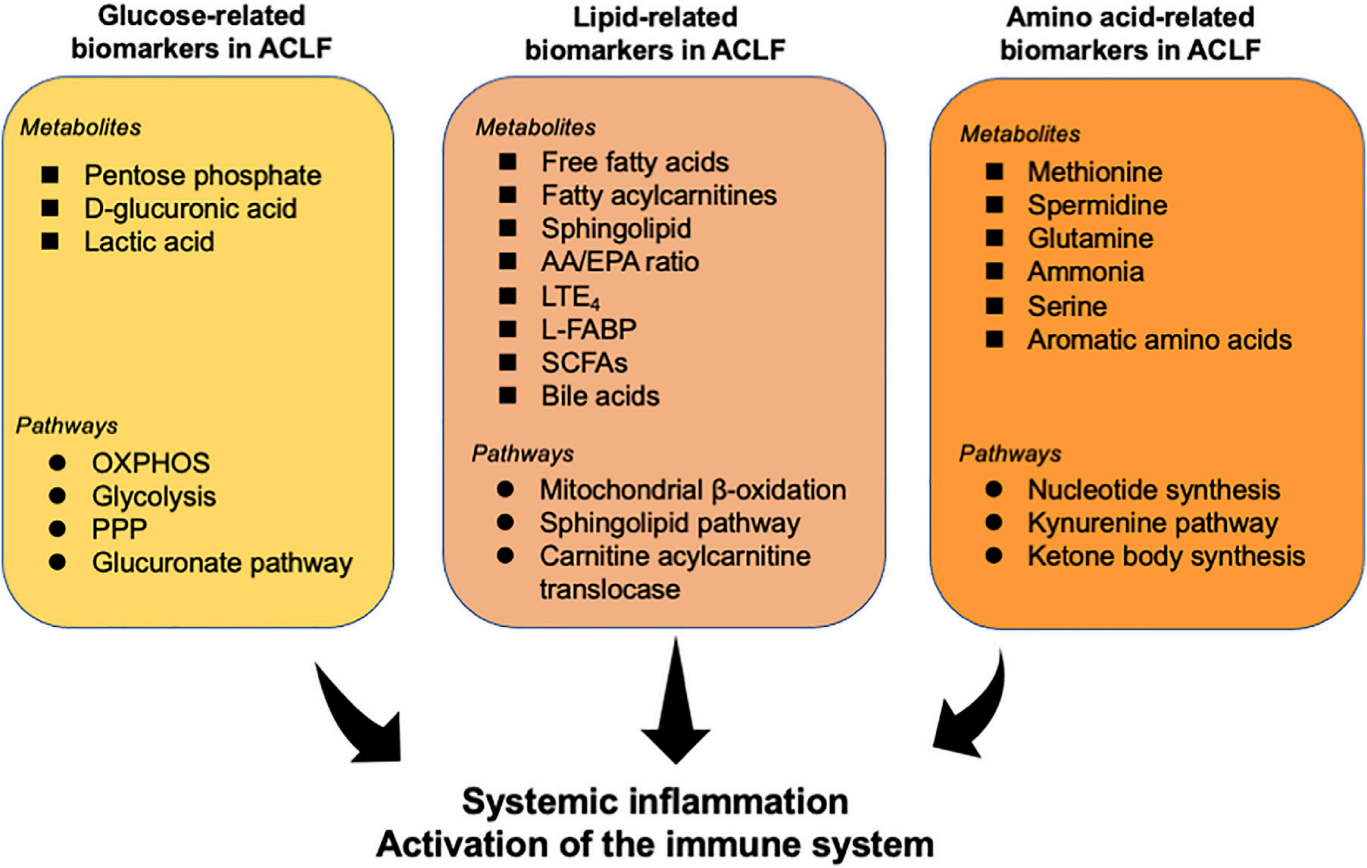
Overview of metabolites and related pathways which have been identified to be robust biomarkers for ACLF. Various metabolites and pathways associated with glycometabolism, lipid metabolism, and amino acid metabolism have been determined to be powerful indicators for ACLF prognosis. These metabolites may be a trigger for the activation of the immune system and systemic inflammation, contributing to the progress of ACLF. AA, arachidonic acid; EPA, eicosapentaenoic acid; L-FABP, liver fatty acid binding protein; LTE_4_, leukotriene E_4_; OXPHOS, oxidative phosphorylation; PPP, pentose phosphate pathway; Short-chain fatty acids (SCFAs).

Recent investigations have determined the alteration of metabolites in patients with ACLF and identified several fingerprints shaped by various metabolites in ACLF (Table 1). Herein, we summarize various key metabolites which are identified to be robust biomarkers for ACLF development (Figure 2).

**TABLE 1:** Biomarkers associated with ACLF from the clinical studies of metabolomics.

First author, year	Sample size	Specimen	Targeted/Untargeted	Technique	Main findings
Moreau, 2020	181 ACLF; 650 AD; 43 CC; 29 HS	Serum	Untargeted	LC-MS	A 38-metabolite cluster including Kynurenic acid, Pentose phosphates, D−Glucuronic acid are significantly associated with ACLF
Bajaj, 2020	602 patients with cirrhosis	Serum	Untargeted	LC-MS	Increased levels of aromatic compounds, secondary or sulfated bile acids, benzoate, and estrogen metabolites, as well as decreased levels of phospholipids, were associated with development of ACLF
López-Vicario, 2020	127 ACLF; 119 AD; 18 HS	Plasma	Targeted lipidomics	LC-MS	LTE4 and 12-HHT, both derived from arachidonic acid, shaped a minimal plasma fingerprint for ACLF
Clària, 2021	Discovery: 518 AD; 43 ACLF Validation: 128 AD; 137 ACLF	Serum	Untargeted lipidomics	LC-MS	Cholesteryl ester and lysophosphatidylcholine composed a fingerprint for ACLF
Pose, 2021	Descriptive cohort: 22 AD; 20 ACLF Intervention cohort: 12 DC treated with simvastatin and rifaximin; 13 DC receiving placebo	Plasma	Untargeted	LC-MS	The signature of 32 metabolites including gluconate is identified to predict the presence of ACLF; Secondary bile acids and dicarboxyl fatty acids decreased in patients treated with simvastatin and rifaximin
Clària, 2019	342 AD; 180 ACLF; 39 CC; 40 HS	Serum; Urine	Untargeted; Targeted	LC-MS	Higher KP activity independently predicted mortality in patients with ACLF
Horvatits, 2016	143 patients with cirrhosis	Serum	Targeted	LC-MS	Serum total and individual BAs are associated with ACLF
McPhail, 2016	Derivation cohort: 43 DC; 37 ACLF Validation cohort: 101 DC; 27 HS	Plasma	Untargeted	H-NMR; LC-MS	Higher levels of lactate, tyrosine, methionine and phenylalanine were found in patients with poor outcome
Amathieu, 2014	30 ACLF; 93 DC	Serum	Untargeted	H-NMR	Increased lactate, pyruvate, ketone bodies, glutamine, phenylalanine, tyrosine, and creatinine shaped a fingerprint for ACLF

ACLF, acute-on-chronic liver failure; AD, acute decompensation; BA, bile acids; CC, compensated cirrhosis; DC, decompensated cirrhosis; 12-HHT, 12-hydroxyheptadecatrienoic acid; H-NMR, proton nuclear magnetic resonance (NMR) spectroscopy; HS, healthy subject; LC-MS, liquid chromatography mass spectrometry; LTE_4_, leukotriene E_4_; KP, kynurenine pathway.

### Biomarkers related to glucose metabolism

In the context of systemic inflammation, ACLF is characterized by a disturbance of energy metabolism. Moreau et al. (Moreau et al., 2020) identified the alterations of serum metabolites associated with glucose metabolism in patients with ACLF through serum metabolomics. In particular, pentose phosphate, as an intermediate of the pentose phosphate pathway, was identified to increase 362-fold in ACLF (which was the strongest alteration in this contrast), 64-fold in acute decompensation, and 16-fold in compensated cirrhosis (CC), compared to healthy subjects (HS), in accordance with previous discoveries in immunometabolism-associated inflammation (O'Neill and Pearce, 2016). The level of d-glucuronic acid increases in ACLF, indicating the increased activity of the glucuronate pathway, which can be connected to the pentose phosphate pathway. Additionally, 4-hydroxy-3-methoxyphenylglycol sulfate, a metabolic product of norepinephrine that can be a stimulant of glycolysis, and blood levels of lactic acid, the key product of glycolysis, also increase in patients with ACLF (Moreau et al., 2020).

### Biomarkers related to the metabolism of amino acids

Skeletal muscle is the largest reservoir of amino acids in the body (Davis and Fiorotto, 2009). High levels of amino acids and peptides likely reflect increased proteolysis and have been associated with a high catabolic status characteristic of inflammatory conditions and sepsis (Hotamisligil, 2017). Similarly, intense proteolysis acids (e.g., tyrosine, asparagine, lysine, methionine, and isoleucine) were increased in serum from patients with ACLF (Moreau et al., 2020; Pose et al., 2021).

Amino acid-related metabolites were associated with intense systemic inflammation and oxidative stress in ACLF and were correlated with organ system failures and severity (Zaccherini et al., 2021). A cluster including 38 metabolites with a remarkably significant association with ACLF was identified via metabonomics analysis combined with bioinformatics analysis and several metabolites of the cluster were amino acids or amino acid derivatives (e.g., saccharopine and N6, N6, N6-trimethyl-l-lysine, the intermediary metabolites in the degradation of lysine) (Moreau et al., 2020). Furthermore, the metabolism of aromatic amino acids, arginine, and benzoates has also been identified to be correlated with the development of ACLF and death (Bajaj et al., 2020).

### Biomarkers related to lipid metabolism

In addition to the alternation of the sphingolipid pathway above-mentioned, bioinformatics analysis of untargeted lipidomics indicated that sphingolipids are the best markers to distinguish acute decompensation from healthy controls and that reduced sphingolipids are associated with the severity of the disease (Clària et al., 2021). A previous investigation revealed that lower serum levels of sphingolipids are associated with worse survival in patients with alcoholic cirrhosis and that impaired sphingolipid biosynthesis is associated with undernutrition in hospitalized patients with AD (Grammatikos et al., 2015; Rachakonda et al., 2019). Furthermore, serum extracellular vesicles loaded with sphingolipids have been shown to predict death in decompensated cirrhosis (Sehrawat et al., 2021). Cholesteryl ester (CE) can accumulate in the adrenal cortex and can be used for the biosynthesis of steroid hormones (Kraemer et al., 2013). Therefore, reduced CE levels could be related to adrenal failure in patients with ACLF (Acevedo et al., 2013). López-Vicario et al. (López-Vicario et al., 2020) uncovered that patients with ACLF had an increased ratio between arachidonic acid and eicosapentaenoic acid, which is an indicator of systemic inflammation (Simopoulos, 2002). Notably, two lipid molecules leukotriene E_4_ (LTE_4_) and 12-hydroxyheptadecatrienoic acid (HHT) were identified to distinguish ACLF from AD. Additionally, it has been proved elevated urinary liver fatty acid binding protein (L-FABP) may suggest the activation of inflammatory pathways through lipid mediators and is correlated with multiorgan dysfunction and serves as a promising biomarker to predict mortality in patients with ACLF (Juanola et al., 2022).

### Biomarkers related to intestinal microbial metabolites

The gut and liver interact through the gut-liver axis and tight bidirectional links through the biliary tract, portal vein, and systemic circulation (Tripathi et al., 2018). Translocation of components or metabolites of the gut microbiota—facilitated by intestinal dysbiosis, increased intestinal permeability and portal hypertension—is a key driver of the development and progression of ACLF (Ridlon et al., 2015; Trebicka et al., 2021). Gut microbiota-associated metabolites can be detected in the serum by blood metabonomics and there are significant changes in the level and composition of metabolites produced by the intestinal microbiota in patients with ACLF (Bajaj et al., 2020; Moreau et al., 2020). The levels of metabolites involved in choline metabolism and most aromatic amino acids decreased in ACLF, while the levels of metabolites associated with tyrosine, secondary bile acids, and benzoate are increased. Of these, 4-methoxyphenol sulfate, which belongs to tyrosine metabolism, is the most correlated with ACLF and is also associated with inpatient death. However, aromatic amino acid metabolites were higher in those who died compared to survivors (Bajaj et al., 2020). Gut microbial metabolites may be valuable biomarkers to identify patients at risk of decompensation and ACLF. However, these signatures are not consistent, and more research is needed.

## Metabolites as potential therapeutic targets for ACLF

With the advent and evolution of metabolomics technologies, the discovery of active metabolites that can change cell physiology has grown rapidly. The active metabolites can regulate phenotypes and pathophysiological processes via interaction with the key proteins or enzymes (Rinschen et al., 2019). Recent investigations have indicated the potential therapeutic benefits of exogenous supplementation of several active metabolites in different liver diseases (Masubuchi et al., 2011; Cai et al., 2016). Mitochondrial dysfunction mediates metabolic disorders in leukocytes in patients with ACLF and may be involved in the progression of organ failure (Zhang et al., 2021). Furthermore, two break points of the TCA cycle in PBMCs were identified: one is the higher utilization of α-KG inducing a reduced utilization of upstream intermediates, including isocitrate, cis-aconitate and citrate; and the other is the higher utilization of fumarate leading to a lower utilization of succinate (Zhang et al., 2021). Therefore, supplementation of the two metabolites may replenish the impaired TCA cycle and attenuate the development of ACLF.

## Conclusion and future directions

This review described the role of metabolomics in investigations aimed at determining biomarkers correlated with the pathophysiology of ACLF. Regarding the high short-term mortality of ACLF, metabolomics should be earlier performed in patients with decompensated cirrhosis, especially in patients presenting with acute variceal bleeding, ascites, or hepatic encephalopathy, to identify individuals at high risk of developing ACLF via target metabolomics, and to enable early preventive interventions. Metabolomics approaches have provided insightful evidence on altered metabolites and metabolic pathways in ACLF. Overall, studies have shown variations in glucose metabolism, amino acid metabolism, several aspects of lipid metabolism, and intestinal microbial metabolism; and identified the association between serum metabolites and the pathogenesis of ACLF. Furthermore, future studies should validate the biomarkers and metabolic pathways reported in the progression of ACLF and explore potential therapeutic targets associated with metabolites for ACLF. It is promising that metabolomics can provide clinical tools for precision therapy for ACLF in the future.

## Abbreviations

ACLF, acute-on-chronic liver failure; PAMPs, pathogen-associated molecular patterns; DAMPs, damage-associated molecular patterns.
